# Enhanced catalytic ozonation of ibuprofen using a 3D structured catalyst with MnO2 nanosheets on carbon microfibers

**DOI:** 10.1038/s41598-021-85651-2

**Published:** 2021-03-18

**Authors:** Guhankumar Ponnusamy, Hajar Farzaneh, Yongfeng Tong, Jenny Lawler, Zhaoyang Liu, Jayaprakash Saththasivam

**Affiliations:** 1 Qatar Environment and Energy Research Institute, Hamad Bin Khalifa University, Qatar Foundation, 34110 Doha, Qatar; 2 Division of Sustainable Development, Hamad Bin Khalifa University (HBKU), Qatar Foundation, 34110 Doha, Qatar

**Keywords:** Chemical engineering, Pollution remediation

## Abstract

Heterogeneous catalytic ozonation is an effective approach to degrade refractory organic pollutants in water. However, ozonation catalysts with combined merits of high activity, good reusability and low cost for practical industrial applications are still rare. This study aims to develop an efficient, stable and economic ozonation catalyst for the degradation of Ibuprofen, a pharmaceutical compound frequently detected as a refractory pollutant in treated wastewaters. The novel three-dimensional network-structured catalyst, comprising of δ-MnO_2_ nanosheets grown on woven carbon microfibers (MnO_2_ nanosheets/carbon microfiber), was synthesized via a facile hydrothermal approach. Catalytic ozonation performance of Ibuprofen removal in water using the new catalyst proves a significant enhancement, where Ibuprofen removal efficiency of close to 90% was achieved with a catalyst loading of 1% (w/v). In contrast, conventional ozonation was only able to achieve 65% removal efficiency under the same operating condition. The enhanced performance with the new catalyst could be attributed to its significantly increased available surface active sites and improved mass transfer of reaction media, as a result of the special surface and structure properties of this new three-dimensional network-structured catalyst. Moreover, the new catalyst displays excellent stability and reusability for ibuprofen degradation over successive reaction cycles. The facile synthesis method and low-cost materials render the new catalyst high potential for industrial scaling up. With the combined advantages of high efficiency, high stability, and low cost, this study sheds new light for industrial applications of ozonation catalysts.

## Introduction

The presence of Ibuprofen in environmental matrices has been attributed to municipal sewage, hospital sewage, pharmaceutical wastewater, and even reclaimed wastewater, and as such Ibuprofen is recognized as a refractory organic pollutant that is recalcitrant to biodegradation process^1,2^. The continuous discharge of ibuprofen into the water environment leads to an irreversible accumulation in the environment, which poses severe risks to human health and the environment^3,4^. It is urgent to develop technologies that can efficiently degrade ibuprofen in the water environment to minimize its risk.

Conventional treatments, based on sedimentation and biological treatment, are not effective for ibuprofen removal^5,6^. Catalytic ozonation, an advanced oxidation process (AOP), has been studied extensively for water treatment, especially for the degradation of refractory organic pollutants in water^7,8^. Some metal oxides, such as MnO_2_, ZnO, Fe_3_O_4_ andTiO_2_, are proven to be promising catalysts in catalytic ozonation by facilitating the generation of free radicals to non-selectively oxidize organic pollutants^9,10^. Both homogenous and heterogeneous catalysts can be used in the ozonation process. However, heterogeneous catalysts are preferred due to the ease of catalyst separation after the process, preventing from secondary pollution^11–13^. MnO_2_ is among the most studied catalysts in water treatment for organic pollutants degradation, due to its cost efficiency and environmental friendliness. The efficiency of catalytic ozonation depends on the textural properties of the catalysts, such as surface area, pore structure, porosity and pore volume^14–16^. With the development of nanotechnology over recent years, nanostructured catalysts have been widely studied for improving the reaction activity^17–19^. Although catalysts with nano/micrometer sizes generally show good performance for catalytic processes, the decreased sizes of the catalysts will lead to high surface energy on the materials, which are prone to agglomeration and lead to a dramatic decrease of their activity^20,21^. Immobilizing metal oxide son porous supports could be effective in inhibiting the agglomeration of these nano/microparticles^22–24^. The performance of cerium oxides supported on activated carbon (CeO_2_-AC) was studied^25^. It was proved that the highly dispersed CeO_2_-AC catalyst could effectively activate ozone (O_3_) conversion into reactive oxygen species (ROS), benefiting from the significantly increased available active sites as well as the facilitated electron transfer between cerium and AC. However, the pore-clogging of porous supports due to metal oxides loading increases the diffusion resistance for mass transfer, which subsequently harms the catalytic performance^26,27^. To tackle the above problems, the development of novel catalysts with ideal textural properties are much needed to achieve the high catalytic activity.

In this study, we designed a novel ozonation catalyst with MnO_2_ nanosheets in situ grown on network-structured woven carbon microfibers (CM). The in situ growing approach enables a strong binding force between MnO_2_ and carbon microfibers substrates, which will render excellent environmental stability and reusability. Moreover, the 3D interconnected porous structure of the new catalyst could offer abundant MnO_2_ catalytic sites and more accessible mass transfer channels with less diffusion resistance. As a result, the novel catalyst is supposed to have superior catalytic activity for Ibuprofen ozonation in liquid media. Simultaneously, to promote the potential for practical industrial applications, a facile method and economic materials were adopted for the synthesis of the catalyst. Altogether, this study expects to provide a new approach for developing ozonation catalyst with combined advantages of high efficiency, high stability and low cost for Ibuprofen degradation.

## Methods

Ibuprofen used in this study was purchased from Sigma Aldrich (China). Ibuprofen stock solution was prepared by dissolving the required amount of ibuprofen in ultra-high quality deionized water (18.2 MΩ) and sonication under ice-cooling for 1 h using 750 W, 20 kHz Cole Parmer sonicator at a pulse 3:1 and 40% amplitude. For all of the experiments, ibuprofen solutions of different concentrations were spiked into the samples using the prepared stock solution.

### Nanomaterial synthesis

**Figure 1:**
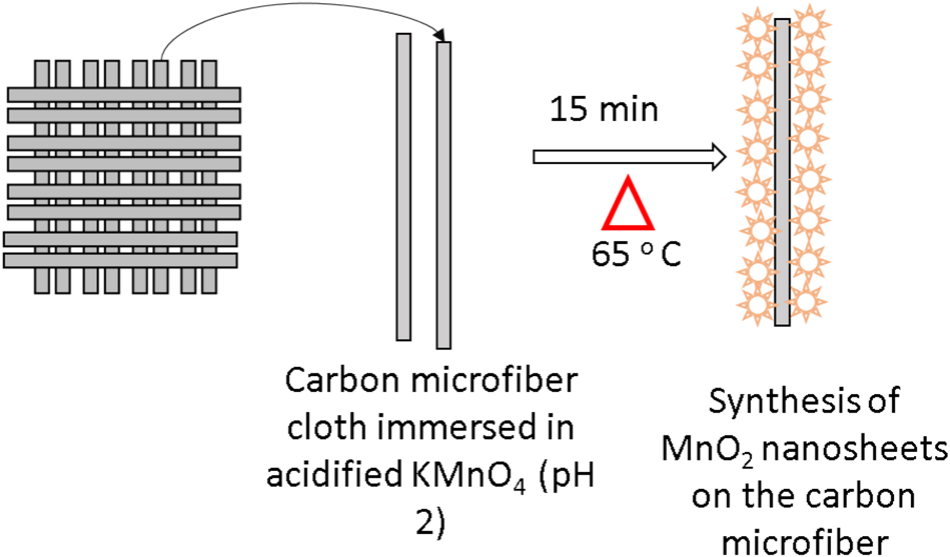
The synthesis process of the new catalyst (MnO_2_ coated carbon microfiber).

MnO_2_ nanosheets were synthesised on pristine carbon microfiber cloths using a facile hydrothermal method. Pristine carbon microfiber cloth with a dimension of 3 cm × 3 cm was washed three times using 50% ethanol and then rinsed with deionised water several times. It was then immersed in potassium permanganate (KMnO_4_) solution at a concentration of (16 g/L) under acidic condition (pH 2) and maintained at 65 °C for 15 min as shown in Fig. 1. The synthesised material was then washed several times using deionised water until all the unspent KMnO_4_ was removed.

### Characterization

The phase composition of the synthesised nanomaterial was characterized using powder X-ray diffraction (XRD). Bruker D8 Advance (Bruker, Germany) with CuKα radiation (λ) 1.540 59 Å) at a step size of 0.02° and a scan rate of 0.05°/min was used to record the results in the 2θ range between 2° and 90°. The X-ray Photoelectron Spectroscopy (XPS) analysis was carried out using standard Thermal-fisher ESCALAB 250XI type XSP platform. A monochromatic Ag Kα Anode X-ray beam is used with a beam current of 14.0 mA, an incident angle of 45° to the sample surface and at an energy of 1486.6 eV. All the spectra obtained were calibrated according to the C–C peak at 284.8 eV in the C1s reference. Infrared spectra were recorded on Thermo Scientific Nicolet iS50 FT-IR spectrometer and the sample discs were prepared in the form of KBr pellets with the sample to KBr ratio 1:100. The FT-IR spectra were recorded with 64 scans per sample and at 4 cm^−1^ resolution over a frequency range of 4000–400 cm^−1^. The chemical composition and surface morphology of the material was studied through field emission scanning electron microscope (FE-SEM, QUANTA FEG 650, Thermo Fisher Scientific, USA) operated with energy dispersive spectroscopy (EDS, Bruker Xflash 6l60, Germany) used for elemental analysis. BET analysis was carried out using Micromeritics ASAP 2020 analyzer (Micromeritics, Norcross, GA). Contact angle measurements were obtained using an advanced goniometer (Rame-hart A100, USA) through a sessile drop method to determine the hydrophilicity of the prepared catalyst. UHPLC (Agilent 1260 Infinity Binary LC, USA) equipped with Acclaim 120, C18, 3 µm Analytical (4.6 × 150 mm) Column (Thermo scientific, USA) was used for the quantification of Ibuprofen. The UHPLC operating conditions used in this study is provided in Table 1^28^.

**Table 1:** UHPLC operating conditions.

Parameters	Conditions
Solvent A	Milli-Q water with 10 mM H_3_PO_4_ (> 85%)
Solvent B	Methanol (≥ 99.9%) HPLC grade
Injection volume (µL)	100
Run time (min)	8
Flow rate (mL/min)	0.8
Temperature (^o^C)	35
Wavelength (nm)	224
Isocratic elution	15% A and 85% B

### Ozonation, ozone-hydrogen peroxide and catalytic ozonation

Ozone was produced using an oxygen-fed 4 g/h corona discharge ozone generator (BMT 802N, Germany). Experiments were performed in 150 mL glass bottles at room temperature and ozone gas from the generator was bubbled directly into the deionized water samples containing Ibuprofen to achieve the required ozone dosage. The concentration of the generated ozone gas was measured using “on-gas” ozone analyzer while the undissolved ozone gas was measured using “off-gas” ozone analyzer. The oxygen flow rate was set at 0.1 Lpm. The residual dissolved ozone in the water was analysed by using the indigo trisulfonate method. For the ozone-based advanced oxidation (AOP) studies, hydrogen peroxide was added and stirred for 1 min before the introduction of ozone gas. For catalytic ozonation experiments, varying weight loadings of the synthesised nanomaterial were added (0.25%, 0.50% and 1.00% (w/v)) to the deionized water samples before ozonation. The equivalent MnO_2_ coated carbon microfiber cloth dimension for 0.25% (w/v), 0.50% (w/v), and 1.00% (w/v) loadings were (1.5 cm × 1.5 cm), (3.0 cm × 1.5 cm) and (3.0 cm × 3.0 cm) respectively. All the above experiments were repeated twice. Samples were withdrawn for ibuprofen quantification at 10, 30, and 60 min time intervals. Residual ozone in the solution was quenched by purging the samples using sodium thiosulphate prior to UHPLC analyses.

## Results and discussion

### Morphology and phase structure characteristics

**Figure 2:**
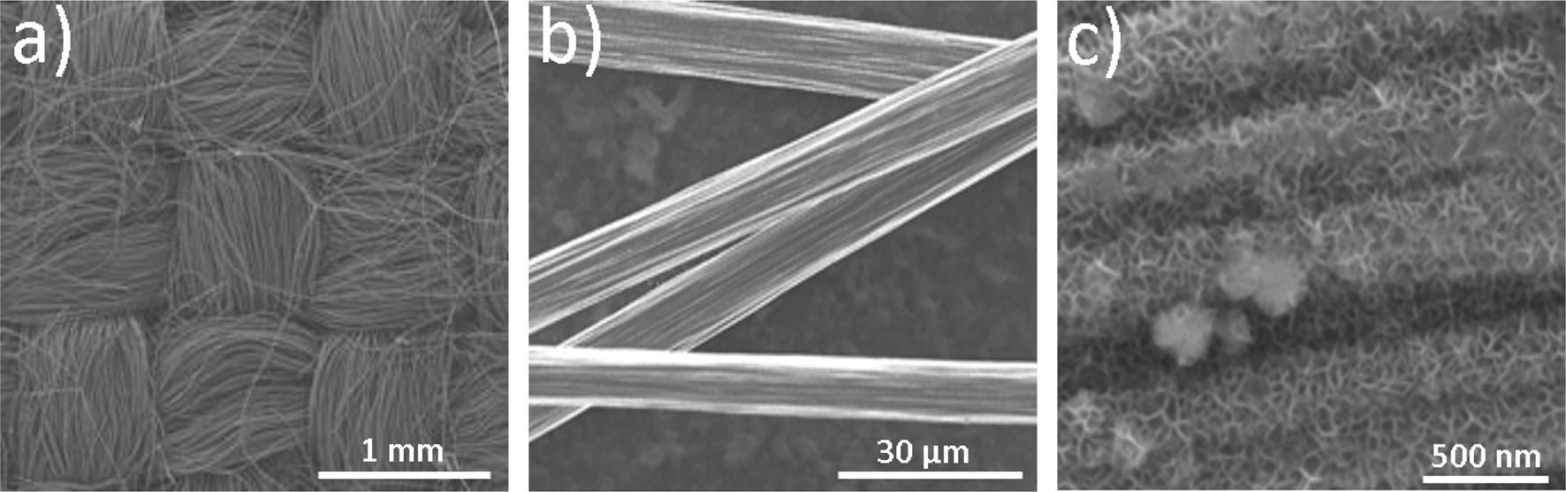
(**a**) SEM image of the pristine woven carbon microfibers, (**b**) SEM image of individual carbon microfibers. (**c**) SEM image of the final product (new catalyst with MnO_2_ nanosheets grown on carbon microfibers).

As shown in Fig. 2a,b, woven carbon cloth (made of carbon microfibers) was chosen here as a catalyst support substrate for MnO_2_ deposition. MnO_2_ nanosheets grown in-situ on the carbon microfibers using a facile hydrothermal method is shown in Fig. 2c. The MnO_2_ nanosheets on carbon microfibers form 3D hierarchical nano/microstructure, which ensures the reaction active sites (MnO_2_ nanosheets) are fully exposed outwards to the reaction media and target pollutants.

**Figure 3:**
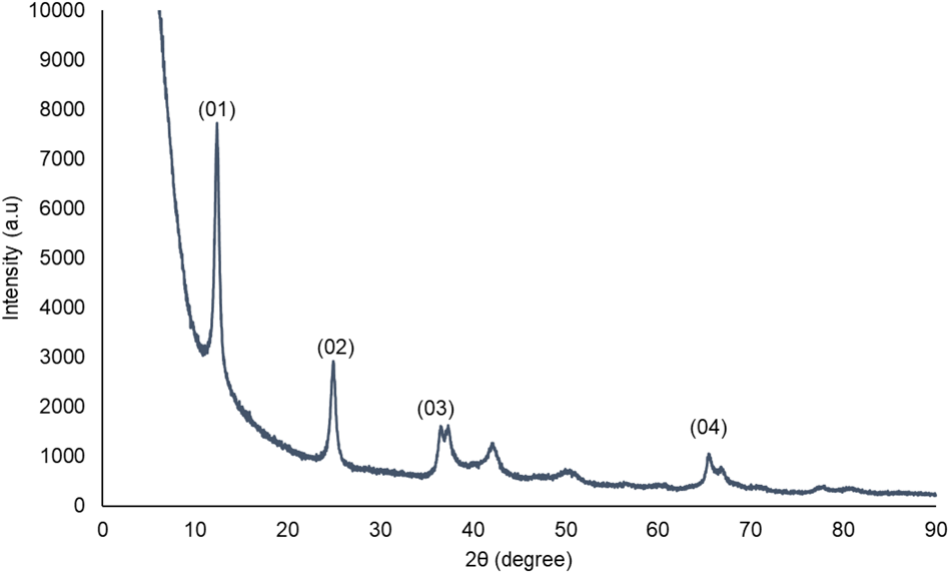
XRD pattern of synthesised MnO_2_.

The XRD profile of the synthesised catalyst is shown in Fig. 3. The diffraction peaks at 2θ of 12.360°, 24.959°, 36.613° and 65.584° were indexed as (01), (02), (03) and (04), respectively. All the observed diffraction peaks match to the phase δ-MnO_2_ (JCPDS 86-0666, SG: R3m, a = 2.849 Å, c = 21.536 Å) or K-birnessite MnO_2_ without characteristic peaks of other phases^29^.

### Physicochemical properties

The chemical composition of the synthesised MnO_2_ nanostructures on carbon microfiber was investigated by IR spectra as shown in Figure S1. More details of the analysis can be found in Figure S1 of the supporting information. SEM–EDS analysis was performed to understand the morphology and to calculate the Mn and O content in the synthesised nanomaterial. The main elements detected on the carbon microfiber surface were Mn and O, illustrating the successful growth of MnO_2_ on the surface of the carbon microfiber. It was found that the final Mn content was 57.08 wt% and oxygen (O) was 30.11 wt%. Figure S2 shows the EDS spectra of the material synthesised having 12.81 wt% of Potassium, which could be due to the usage of KMnO_4_ as a precursor.

**Figure 4:**
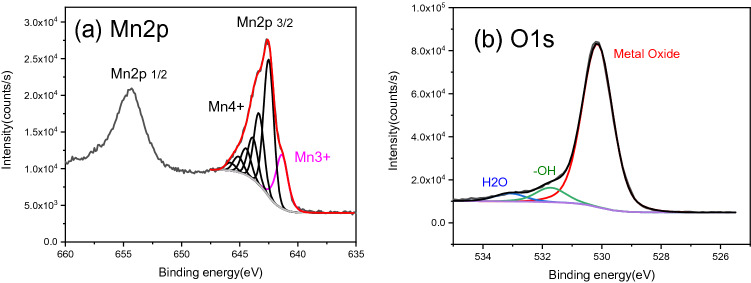
Mn2p (**a**) and O1s (**b**) XPS core level spectra of the synthesised MnO_2_ nanomaterial. The spectra are fitted after a proper Shirley background subtraction. The Mn2p 3/2 component is fitted due to the multiple splitting and the corresponding overlapping in binding energy ranges. The pink curve indicates the Mn3 + and the rest 6 black curves represent the Mn4 + oxidation state. O1s was fitted with three components corresponding to the Metal oxide, the –OH and the H_2_O.

In order to reveal the oxidation information of Mn, a deconvolution of the Mn2p and O1s is applied after a proper Shirley background subtraction. Particularly in the Mn2p case, only the Mn2p 3/2 component was fit due to the fact that the transition metal contains strong variation in both the splitting energy position and the intensity ratio between the two splitting orbitals^30^. As indicated in Fig. 4a, the spectrum of Mn2p appears very similar to what has been reported in MnO_2_ films^31^ and no obvious MnO related shake-up satellite is observed at 647 eV. Thus a similar fitting method was applied which resulted in a small quantity (18.4%) of Mn3 + at 642 eV and a majority (81.6%) of the Mn4 + at higher binding energy range, which is represented by six black components. This is in coordinate with what is observed in the XRD results where a main crystalline phase of MnO_2_ is observed. Accordingly, a strong metal oxidation state is given in the O1s spectrum in Fig. 4b at 530.0 eV and small-signal of (–OH) and H_2_O related structure can also be located at 531.7 eV and 533.0 eV^32^. The Stoichiometry is thus estimated to be around MnO_1.8_(OH)_0.18_ by considering the intensity of the Mn2p doublet and the metal oxide feature in the O1s. The formation of ·OH radical and its substitution has been recently proposed as the catalyst mechanism for this MnOx(OH) system^33^. It involves the attachment of H_2_O through the oxygen vacancy of MnOx materials surface and further releasing of ·OH radicals and O_2_ when meeting the O_3_ target. In the present case, Mn4 + state in our MnO_2_ materials is proved by both the XRD and XPS (both deconvolution and stoichiometry estimation); while the presence of (–OH) structure is confirmed with the observation of the O–H bond bending vibration mode and the correlated component at 531.7 eV in the O1s XPS analysis. The total stoichiometry of the material turned out to be very similar to the Birnessite (MnO_1.75_(OH)_0.25_), whose surface presents a strong capability of oxidizing a wide range of organic/inorganic compounds^34^.

**Figure 5:**
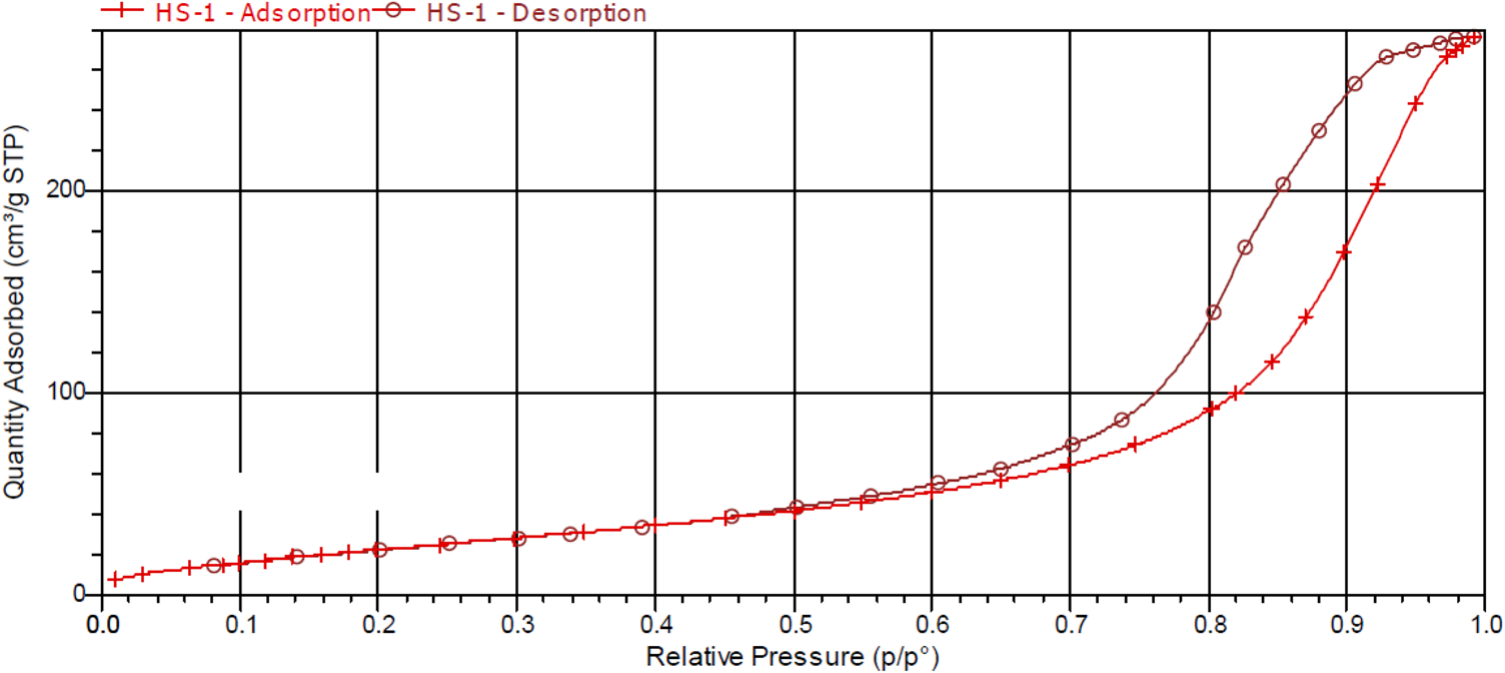
Nitrogen adsorption–desorption isotherms of the new catalyst with MnO_2_ nanosheets grown on carbon microfibers.

The catalytic performance of the material is mainly affected by the specific surface area and the relative distribution of pore size on the material synthesised. Measurements were taken using the Brunauer–Emmett–Teller (BET) method for the pristine carbon microfibers and the MnO_2_ coated carbon microfibers. The adsorption–desorption isotherms shown in Fig. 5 indicates type IV isotherm with the formation of H3 hysteresis loop, with values in the middle range of 0.5 < P/P0 < 1.0^35^_._ The low BET surface area of the pristine material, 0.7026 m^2^ g^−1^ was similar to the value reported in another study that used carbon fibers for the synthesis of carbon nanomaterials^36^. Nitrogen adsorption–desorption isotherm of the pristine carbon microfibers is shown in Figure S3 of the supporting information. For the MnO_2_ coated carbon microfibers, the calculated BET surface area and mean pore diameter were 97.2698 m^2^ g^−1^ and 9.75 nm respectively. The BET surface area for the synthesised catalyst is higher than the pristine carbon microfibers, which is due to the formation of MnO_2_ nanomaterials on the surfaces of the carbon microfibers. This high surface area of new catalyst suggests the high amount of MnO_2_ catalytic active sites are fully exposed and accessible for the catalytic reaction.

**Figure 6:**
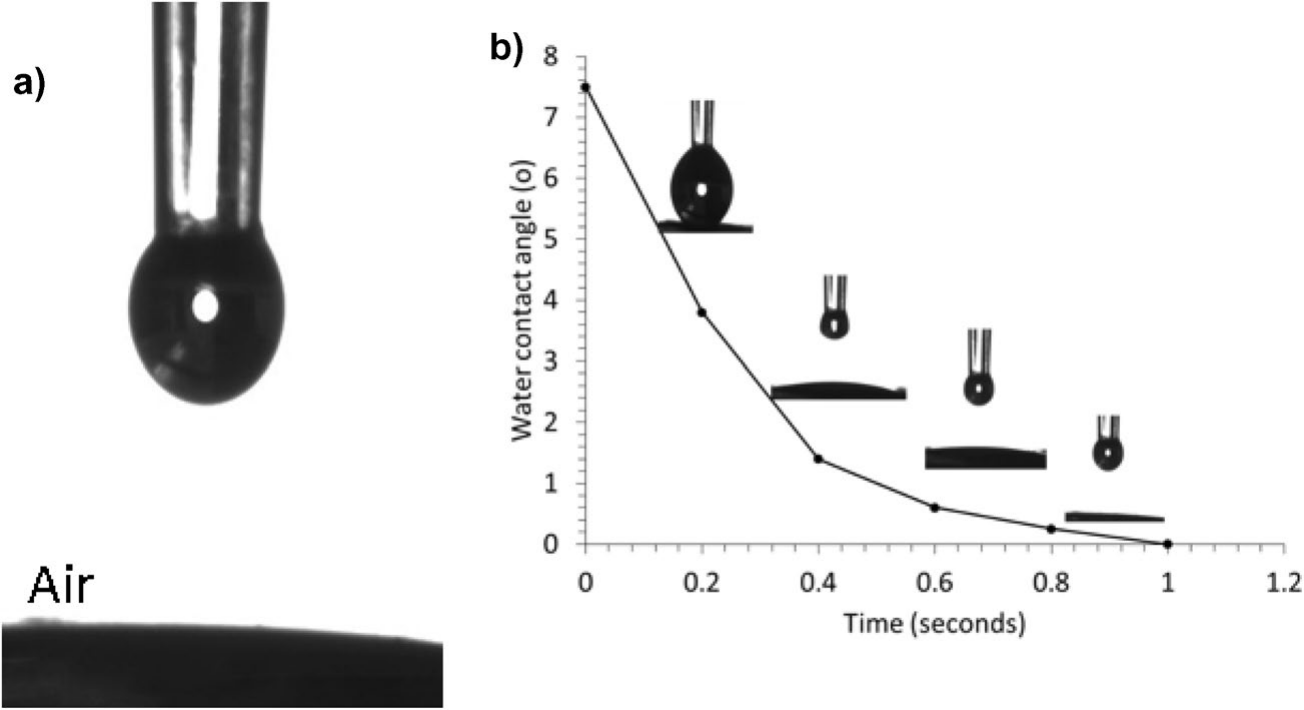
Water contact angle measurement of the MnO_2_ coated carbon microfiber using a sessile drop method. (**a**) Water contact angle of the MnO_2_ coated on the carbon microfibers. (0°) and (**b**) dynamic water contact angles: water droplet behaviour when water drops on the carbon microfibers coated with MnO_2_ nanosheets. Water contact angle measurement for pristine carbon microfiber can be found in Figure S4 of the supporting information.

As provided in Fig. 6a, water contact angle on the new catalyst withMnO_2_ nanosheets grown on carbon microfibers was zero, thus indicating the super-hydrophilicity of the membrane. Another interesting phenomenon is that the water droplet swiftly sinks into the catalyst substrate within less than one second, as shown in Fig. 6b, which strongly suggests the fast mass transfer for water-based reaction media. This is due to the new catalyst’s special surface and structure properties, including (1) its hydrophilic surface that is fully covered by hydrophilic MnO_2_, and (2) its three-dimensional interconnected porous structure that is rendered by woven fabric substrate. The fast mass transfer is a favourable factor for a catalyst to ensure fast reaction kinetics.

### Performance of new catalyst on ibuprofen degradation

Adsorption experiments were carried out at different catalytic loadings (0.25% (w/v), 0.50% (w/v), and 1.00% (w/v)) with an initial ibuprofen concentration of 5 mg/L for a period of 24 h. Changes in the concentrations were measured by taking samples and analyzing at varying time intervals. Figure S5 clearly shows that the adsorption efficiency of ibuprofen at these three different loadings was poor. A maximum of 17% ibuprofen removal efficiency was achieved using the highest catalyst loading of 1.00% (w/v). The poor removal efficiency could be due to the non-adsorption of the organics on the surface of the catalyst^37^. The pH of the bulk solution and pH point of zero charge (pH_PZC_) of the catalyst also plays a major role during the adsorption process. As reported in previous studies using electrophoretic mobilities and Na + and K + adsorption, pH_PZC_ of δ-MnO_2_ is found to be within the range of 2.25–2.8^38^. The reported pK_a_ values for ibuprofen at 25 °C and 37 °C is 4.3 even at the maximum ionic strength of 0.12 mol/L^39^. Therefore, ibuprofen mainly exists in anionic form while the surface of the MnO_2_ is expected to be negatively charged within the pH range used in this study. This could lead to higher electrostatic repulse force that possibly limits the adsorption of ibuprofen towards the surface of the catalyst. To investigate the removal of non-adsorbed organics from the bulk solution, heterogeneous catalytic ozonation studies were carried out using the synthesised catalyst.

**Figure 7:**
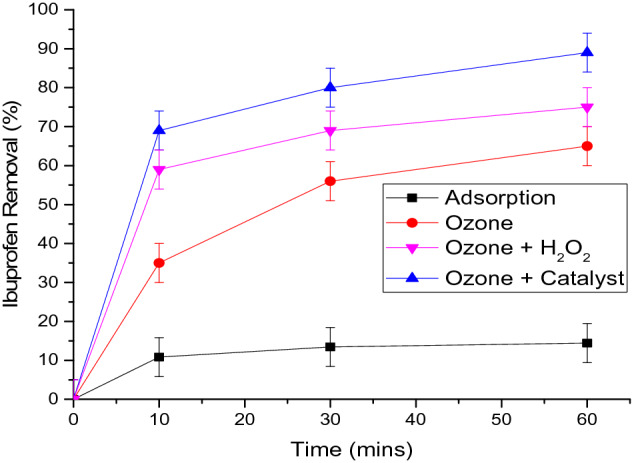
Ibuprofen removal using different treatment methods. Experimental conditions: pH—6.5, temperature 20 ± 1 °C, initial Ibuprofen concentration—5 mg/L, catalyst—1.00% (w/v), ozone dose—2 mg/L, H_2_O_2_ dose—0.5 mg/L.

Figure 7 compares the efficiency of ibuprofen removal through adsorption, conventional ozonation, ozone-hydrogen peroxide (peroxone) and catalytic ozonation using the synthesised material. As explained earlier, adsorption showed low removal of the ibuprofen during the one hour treatment period. Conventional ozonation removed nearly 65% of ibuprofen while peroxone showed an additional removal efficiency of 10% at an ozone dosage of 2 mg/L after one hour of reaction. Results of the ozonation experiments with varying ozone dosages for ibuprofen removal is provided in Figure S6 of the supporting information document with an explanation for choosing 2 mg/L ozone dosage in the subsequent experiments. In the ozonation process, both molecular ozone and ˙OH radicals can be involved in the degradation of organics where the degradation route is highly dependent on the chemical structure of the targeted compound. Addition of H_2_O_2_ in the O_3_/H_2_O_2_ process enhances the formation of ˙OH radicals, a much stronger oxidant than molecular ozone, which serves as the core active species^40,41^. As illustrated in Fig. 7, more ibuprofen was removed by O_3_/H_2_O_2_ compared to the ozonation alone which is due to the higher and faster generation of ˙OH radicals. This is in agreement with other studies where the main pathway for ibuprofen removal was found to be through ˙OH radicals with limited removal by molecular ozone^42–44^. Ozonation of ibuprofen using only the pristine carbon cloth exhibited almost similar results as the conventional ozonation experiments. This indicates that carbon microfiber did not play an active role in the removal of ibuprofen. On the other hand, it can be seen that the best removal efficiency was achieved by using the MnO_2_ nanosheets grown on carbon microfibers where nearly 89% of ibuprofen was removed within an hour. It is also important to note that the catalyst was able to remove 70% of the ibuprofen within the first 10 min of reaction while the removal efficiency for adsorption and ozonation were only 11% and 35% respectively. This proves that the synthesized catalyst acts as a viable material for catalytic ozonation as it provides abundant active reaction sites for catalysis process and fast mass transfer due to its 3D porous structure and super hydrophilicity. The formation of ˙OH radicals due to adsorption and decomposition of ozone on the surface of the catalyst could be one of the reasons behind the higher removal efficiency. In addition, the degradation efficiency could also be boosted by peroxide species (O_2_*) that are formed due to the decomposition of ozone on the active oxygen vacancy site of MnO_2_^45,46^. Table 2 compares the findings of this study against other advanced oxidation processes used to remove ibuprofen.

**Table 2:** Comparison of Ibuprofen removal using different catalytic ozonation process.

Catalyst	Ibuprofen conc. (mg/L)	Removal (%)	Other details	References
γ-Alumina	15	83	pH = 7.2 time = 30 min Ozone = 0.5 mg/min	^47^
GO/Fe_3_O_4_	0.1	85	pH = 7.0 time = 5 min Ozone = 4 mg/L	^48^
γ-Ti-Al_2_O_3_	10	100	pH = 7.0 time = 20 min	^49^
δ-MnO_2_	10	10	time = 20 min Ozone = 10 mg/L	^50^
MnO_2_ coated carbon microfiber	5	89	pH = 6.5 time = 60 min Ozone = 2 mg/L	Present study

### Hydroxyl radical investigation

**Figure 8:**
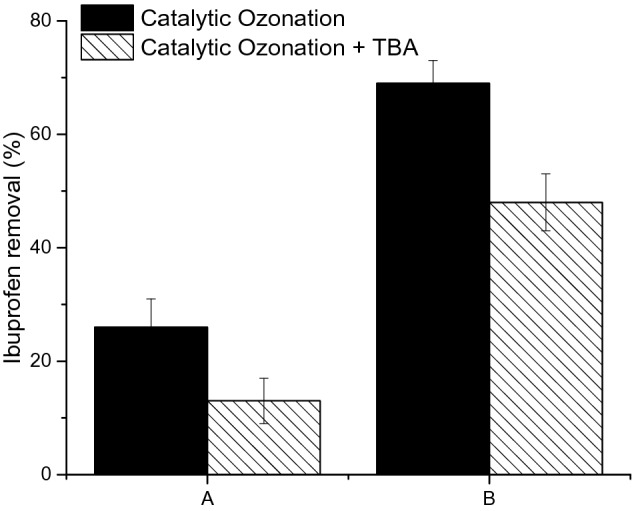
Effect of TBA on the removal of Ibuprofen using catalytic ozonation. Experimental conditions: pH—6.5, temperature 20 ± 1 °C, initial Ibuprofen concentration—5 mg/L, catalyst—1.00% (w/v), time—10 min, A: ozone dose—1 mg/L, TBA—10 mg/L, B: ozone dose—2 mg/L, TBA—20 mg/L.

In order to confirm the contribution of the radical to ibuprofen degradation, tertiary butyl alcohol (TBA) was used to scavenge the radicals that are produced during the catalytic ozonation reaction^51,52^. The rate constant of TBA with hydroxyl radicals and ozone is 5 × 10^8^ M^−1^ s^−1^ and 3 × 10^–3^ M^−1^ s^−1^, respectively^29^, hence TBA is an excellent free hydroxyl radical scavenger. As shown in Fig. 8, when TBA was added to the catalytic reaction, the removal rate of ibuprofen during the first 10 min of reaction was lower than the trials without TBA. This indicates that the generation of hydroxyl radicals do contribute to the degradation of ibuprofen as mentioned earlier.

### Effect of catalyst loading and pH

**Figure 9:**
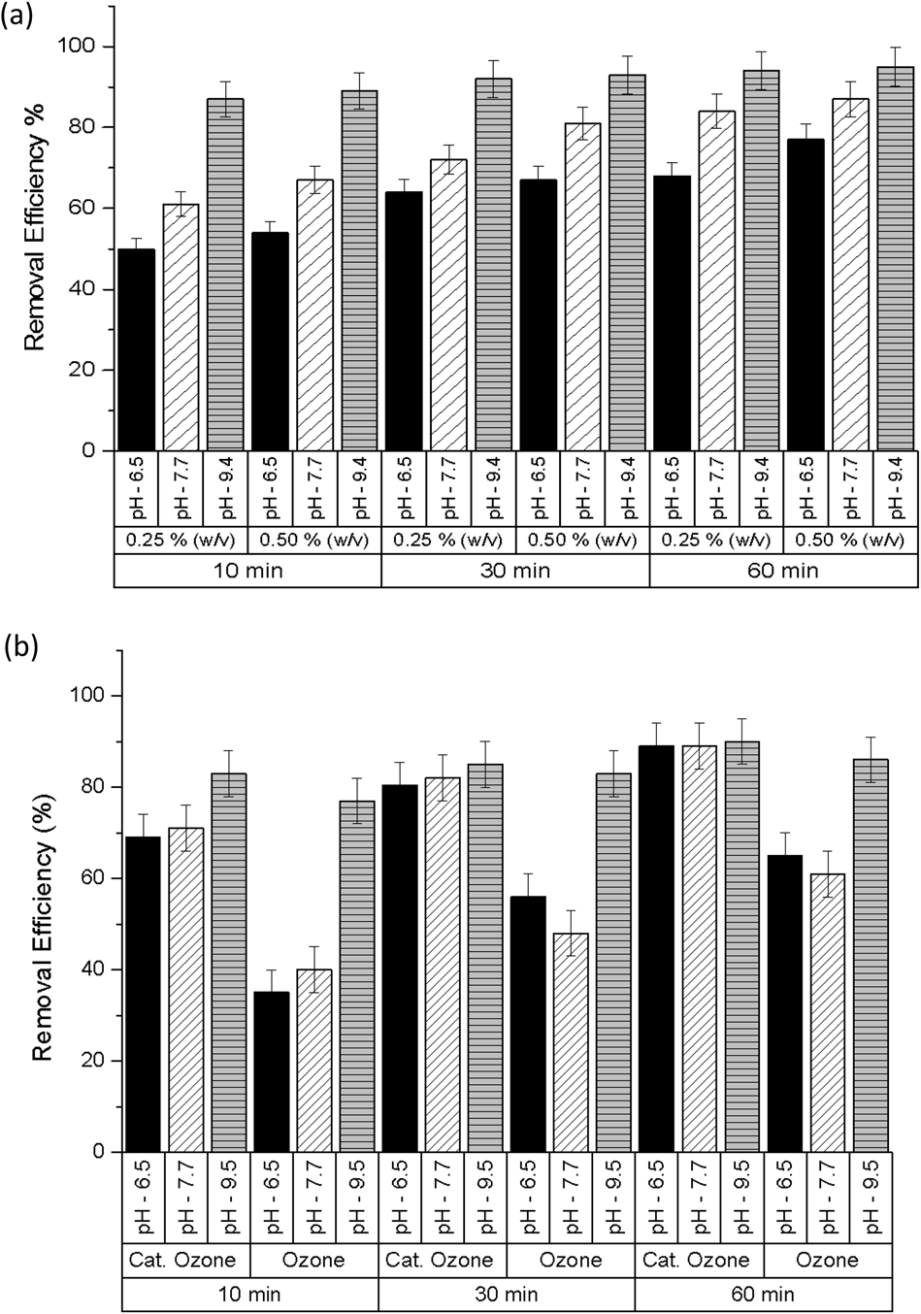
(**a**) Ibuprofen removal at varying pH and different catalyst load. Experimental conditions: temperature 20 ± 1 °C, initial Ibuprofen concentration—5 mg/L, pH 6.5, 7.7 and 9.4, catalyst load—0.25% and 0.50% (w/v), ozone dose—2 mg/L. (**b**) Ibuprofen removal at varying pH conditions. Experimental conditions: pH—6.5, 7.7, and 9.5, temperature 20 ± 1 °C, initial Ibuprofen concentration—5 mg/L, catalyst—1.00% (w/v), ozone dose—2 mg/L.

The effects of catalyst loading and pH on the ibuprofen removal were also investigated in this study where varying amounts of catalyst (0.25% and 0.50% (w/v)) were tested at different pH (low—6.5, medium—7.7, high—9.5). From Fig. 9a,b, it is clear that ibuprofen removal efficiencies increased with catalyst loading. Higher loading provides more active surface sites that are required for the catalytic ozonation process. Also, higher pH contributes to the better removal rate of ibuprofen. Apart from the formation of various radicals on the surface of the catalyst, higher pH also led to quicker decomposition of ozone to ˙OH radicals that could lead to better ibuprofen removal efficiency. Additional experiments were carried out at catalyst loading of 1.00% (w/v) to compare the efficiency of conventional ozonation and catalytic ozonation at various pH levels. From Fig. 9b, it can be seen that the removal efficiency of conventional ozonation at pH 6.5, 7.7 and 9.5 were 65%, 61%, and 89.5% respectively. As explained earlier, the significant increase in the removal efficiency at pH 9.5 could be due to a higher decomposition rate of the ozone at increasing pH to form ˙OH radicals^53^. In contrast, ibuprofen removal efficiency as high as 89% was achieved after one hour of reaction even at pH 6.5 during the catalytic ozonation process.

### Reusability of catalyst

**Figure 10:**
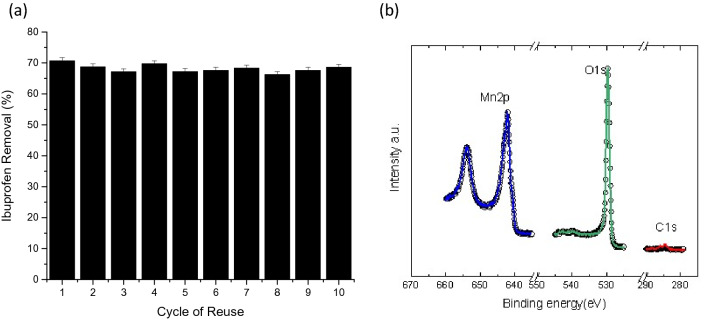
(**a**) Ibuprofen removal efficiency of the catalyst after several cycles of reuse. Experimental conditions: pH—6.5, temperature 20 ± 1 °C, initial Ibuprofen concentration—5 mg/L, catalyst—1.00% (w/v), time—10 min, ozone dose—2 mg/L. (**b**) Catalyst stability by comparing the Mn2p, O1s and C1s core level before (solid line ) and after (dot line) catalytic ozonation. A normalization in intensity was given.

The reusability of the MnO_2_ coated carbon microfiber catalyst in consistently removing ibuprofen was also included in this study. After each cycle, the catalyst was collected, rinsed with deionized water several times and dried at 50 °C before its consecutive reuse. Figure 10a shows that the catalyst was able to consistently remove close to 70% of ibuprofen throughout the 10 cycles of reuse within the first 10 min of reaction. The stability of the catalyst was also confirmed in this study by comparing O1s and Mn2p before and after the catalytic ozonation process. As shown in Fig. 10b, the overlap between the solid line (before ozonation) and dotted line (post ozonation) indicates that the MnO_2_ structure is identical and stable. Particularly, the C1s region intensity is very low which signifies poor adsorption of the intermediaries on the synthesized nanomaterial even after successive usage. Therefore, the application of the synthesised catalyst was feasible for water treatment since it can be easily recovered by being intact on the surface of the carbon microfiber cloth. As the effect of adsorption on the catalyst is minimal with lower contamination potential, hence the catalyst can be effectively reused for several times.

## Conclusions

Catalytic ozonation was found to be an effective treatment method for the removal of ibuprofen which is a persistent pharmaceutical compound to the conventional water and wastewater treatment methods. The adsorption effect of the synthesized catalyst was found to be very low with less than 20% ibuprofen removal at the studied experimental conditions. The removal of ibuprofen was also studied by ozone alone and O_3_/H_2_O_2_ where the removal efficiency by ozonation was found to be about 65% and with the addition of H_2_O_2,_ the removal efficiency was increased by 10%. Ibuprofen is mainly oxidized by ˙OH radicals rather than the molecular ozone which is the reason for the enhanced removal efficiency in the O_3_/H_2_O_2_. This was also confirmed in this study by adding TBA, a strong ˙OH radical scavenger, during catalytic ozonation test. The maximum removal efficiency of ibuprofen of around 90% was achieved by catalytic ozonation where a vast majority of ibuprofen was removed in the first 10 min of reaction. The successful reusability test of the catalysts within 10 cycles shows that they can be reused several times with high efficiencies comparable to the fresh catalysts. These tests conclude that the synthesized catalysts can be used in larger scales for catalytic ozonation water treatment processes with longer catalyst life and high efficiency.

## Supplementary information

Supplementary information.
