# Supplementary material for: Enhanced catalytic ozonation of ibuprofen using a 3D structured catalyst with MnO2 nanosheets on carbon microfibers
Source: Sci Rep. 2021 Mar 18;11:6342. doi: 10.1038/s41598-021-85651-2 (PMC7973777; doi:10.1038/s41598-021-85651-2)
Supplement: Supplementary file 1 — Supplementary information. [file 41598_2021_85651_MOESM1_ESM.docx]

**Supporting Information**

**Enhanced catalytic ozonation of ibuprofen using a 3D structured catalyst with MnO2 nanosheets on carbon microfibers**

Guhankumar Ponnusamy^1^, Hajar Farzaneh^2^, Yongfeng Tong^1^, Jenny Lawler^1^, Zhaoyang Liu^1*^, and Jayaprakash Saththasivam^1*^,

^1^Qatar Environment and Energy Research Institute, Hamad Bin Khalifa University, Qatar Foundation, Doha, 34110, Qatar

^2^Division of Sustainable Development, Hamad Bin Khalifa University (HBKU), Qatar Foundation, Doha, 34110, Qatar

Corresponding author email: [zhliu@hbku.edu.qa](mailto:zhliu@hbku.edu.qa);[jsaththasivam@hbku.edu.qa](mailto:jsaththasivam@hbku.edu.qa)

**S1: FTIR analysis**

FTIR analysis of the synthesized MnO_2_ nanomaterial is provided in Figure S1. The broad absorptions observed at 3445 cm^-1^ is assigned to the stretching vibrations of –OH group of adsorbed water, while that at 1630 cm^-1^ is due to the bending vibration of –OH group. The sharp vibration at 520 cm^-1^ was contributed by the vibrations of Mn–O bonds. From the above results, it is confirmed that the sharp peak below the wavelength of 1000 cm is due to the inter-atomic vibrations caused by the metal-oxide bond.


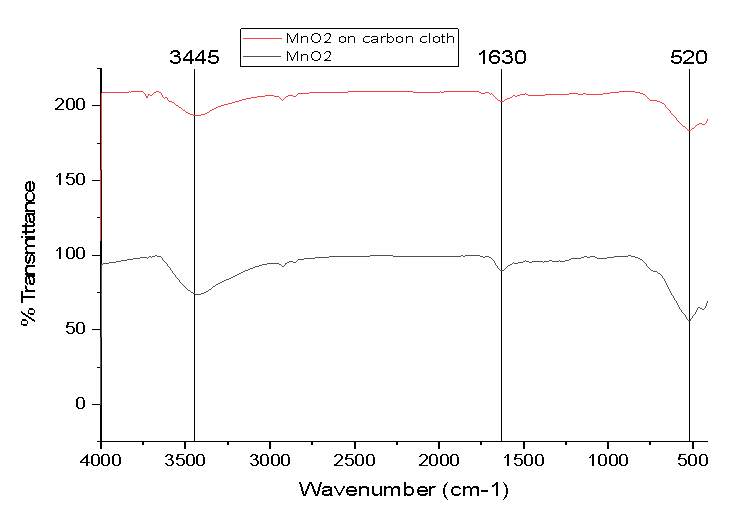


Figure S1. FTIR pattern of synthesised MnO_2_

**S2: SEM-EDS analysis**

SEM-EDS analysis was performed to understand the morphology and to calculate the Mn and O content in the synthesised nanomaterial. The main elements detected on the carbon microfiber surface were Mn and O, illustrating the successful growth of MnO_2_ on the surface of the carbon microfiber. It was found that the final Mn content was 57.08 wt% and oxygen (O) was 30.11 wt%. Figure S2 shows the EDS spectra of the material synthesised having 12.81 wt% of Potassium, which could be due to the usage of KMnO_4_ as a precursor.


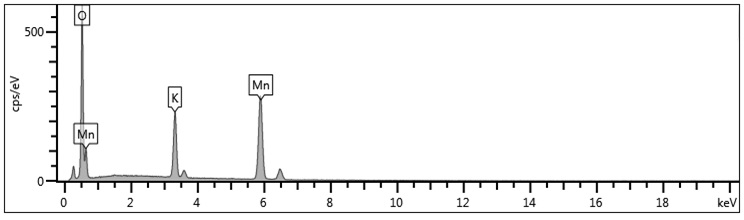


Figure S2. EDS analysis showing the purity of synthesised MnO_2_ nanomaterial

**S3: Nitrogen adsorption-desorption isotherm of the carbon microfibers**

**
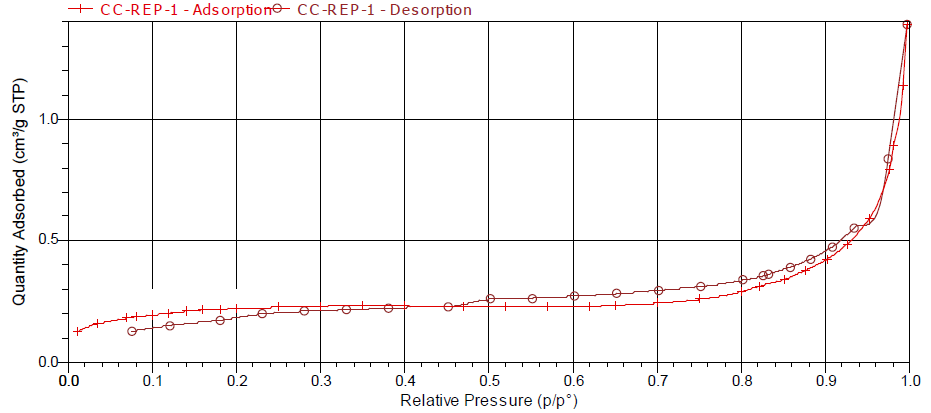
**

Figure S3. Nitrogen adsorption-desorption isotherm of the carbon microfibers.

The adsorption-desorption isotherm for carbon microfibers shown in Figure S3 indicates type IV isotherm with the formation of the H4 hysteresis loop, with values in the middle range of 0.464<P/P0<1.0. The calculated BET surface area and mean pore diameter were 0.7026 m^2^ g^-1^ and 12.72 nm respectively.

**S4: Water contact angle profile of pristine carbon microfiber**

The surface wettability of the carbon microfiber substrate was studied by water contact angle measurement. Figure S4 shows a water contact angle of 135 ^o^C on the carbon microfiber surface, which indicates the highly hydrophobic property of these carbon microfibers.


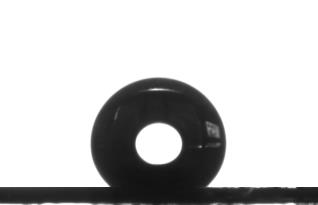


Figure S4.Water contact angle profile of pristine carbon microfiber using the sessile drop method.

**S5: Adsorption of Ibuprofen at different catalytic loadings**

Figure S5. Adsorption of Ibuprofen at different catalytic loadings. Experimental conditions: Temperature 20 ± 1 ^o^C, Initial ibuprofen concentration – 5 mg/L, pH – 6.5, catalyst load – 0.25%, 0.50% and 1.00% (w/v)

**S6: Ibuprofen removal at varying ozone dosage**

Figure S6. Ibuprofen removal at varying ozone dosage. Experimental conditions: pH – 6.5, Temperature 20 ± 1 ^o^C, Initial Ibuprofen concentration – 5 mg/L, catalyst – 1.00% (w/v), Time – 60 min A: Ozone dose – 1 mg/L, B: Ozone dose – 2 mg/L and C: Ozone dose – 5 mg/L

Ibuprofen removal efficiency at different ozone dosages is shown in Figure S6. It can be seen that ozone dosage plays an important role in the ibuprofen removal process for both conventional and catalytic ozonation processes. At 1 mg/L ozone dose, the removal efficiency was low in both ozonation and catalytic ozonation. This is due to the insufficient amount of the oxidant and radicals required for the oxidation of high concentration of Ibuprofen. In the case of 5 mg/L ozone dosage, both ozonation and catalytic ozonation showed similar removal efficiency up to 95% due to the higher ozone to ibuprofen ratio. The advantage of catalytic ozonation can be seen at 2 mg/L ozone dose where ibuprofen removal efficiency of catalytic ozonation was 24% higher than conventional ozonation. Subsequent experiments related to catalytic ozonation in this study were carried out at 2 mg/L ozone dosage to further investigate the performance of the synthesised catalyst.

**S7: Residual dissolved ozone profile**

Figure S7. Residual dissolved ozone profile. Experimental conditions: pH – 6.5, Temperature 20 ± 1 ^o^C, Initial Ibuprofen concentration – 5 mg/L, catalyst – 1.00% (w/v), A: Ozone dose –Ozone dose – 2 mg/L
